# Measurement accuracy and reliability of tooth length on conventional and CBCT reconstructed panoramic radiographs

**DOI:** 10.1590/2176-9451.19.5.045-053.oar

**Published:** 2014

**Authors:** Carlos Flores-Mir, Mark R Rosenblatt, Paul W. Major, Jason P. Carey, Giseon Heo

**Affiliations:** 1 Associate professor and head of the Department of Orthodontics, University of Alberta; 2 MSc in Orthodontics, University of Alberta; 3 Chair of the Department of Dentistry, Faculty of Medicine and Dentistry, University of Alberta; 4 Associate professor, Department of Mechanical Engineering, Faculty of Engineering, University of Alberta; 5 Associate professor of Statistics, Department of Dentistry, University of Alberta, Edmonton, Alberta, Canada

**Keywords:** Reproducibility of results, Radiography, Tooth root

## Abstract

**INTRODUCTION:**

This *in vivo* study assessed accuracy and reliability of tooth length measurements obtained from conventional panoramic radiographs and CBCT panoramic reconstructions to that of a digital caliper (gold standard).

**METHODS:**

The sample consisted of subjects who had CBCT and conventional panoramic radiographic imaging and who required maxillary premolar extraction for routine orthodontic treatment. A total of 48 teeth extracted from 26 subjects were measured directly with digital calipers. Radiographic images were scanned and digitally measured in Dolphin 3D software. Accuracy of tooth length measurements made by CBCT panoramic reconstructions, conventional panoramic radiographs and digital caliper (gold standard) were compared to each other by repeated measures one-way ANOVA with Bonferroni correction and by single measures intraclass correlation coefficient.

**RESULTS:**

Repeated root length measures with digital calipers, panoramic radiographs and CBCT constructed panoramic-like images were all individually highly reliable. Compared to the caliper (gold standard), tooth measurements obtained from conventional panoramic radiographs were on average 6.3 mm (SD = 2.0 mm) longer, while tooth measurements from CBCT panoramic reconstructions were an average of 1.7 mm (SD = 1.2 mm) shorter.

**CONCLUSIONS:**

In comparison to actual tooth lengths, conventional panoramic radiographs were relatively inaccurate, overestimating the lengths by 29%, while CBCT panoramic reconstructions underestimated the lengths by 4%.

## Abstract (pt)

**INTRODUÇÃO:**

este estudo *in vivo* avaliou a precisão e a confiabilidade de medições do comprimento dentário realizadas em radiografias panorâmicas convencionais e em reconstruções panorâmicas de tomografias computadorizadas de feixe cônico (TCFC), comparando-as com medições feitas com um paquímetro digital, consideradas o padrão-ouro.

**MÉTODOS:**

a amostra incluiu indivíduos que já tivessem realizado tanto exames imaginológicos de TCFC quanto radiografias panorâmicas, e cujo tratamento ortodôntico exigisse a extração de pré-molar superior. No total, 48 dentes extraídos, de 26 pacientes, foram mensurados diretamente com paquímetros digitais. As radiografias foram escaneadas e digitalmente avaliadas com a ajuda do *software* Dolphin 3D. Por meio da análise de variância simples com correção de Bonferroni e Coeficiente de Correlação Intraclasse simples, comparou-se a precisão das medições de comprimento dentário realizadas em reconstruções panorâmicas de TCFC, em radiografias panorâmicas convencionais e com paquímetro digital.

**RESULTADOS:**

medições repetidas de comprimento dentário feitas com o paquímetro digital, radiografias panorâmicas e reconstruções panorâmicas de TCFC foram todas consideradas, individualmente, altamente confiáveis. Em comparação ao paquímetro, as medidas obtidas por meio de radiografias panorâmicas convencionais foram, em média, 6,3 ± 2,0mm mais longas, enquanto as medidas obtidas por meio das reconstruções panorâmicas de TCFC foram, em média, 1,7 ± 1,2mm mais curtas.

**CONCLUSÕES:**

em comparação com o real comprimento dentário, as radiografias panorâmicas convencionais foram relativamente imprecisas e superestimaram o comprimento em 29%; já as reconstruções panorâmicas de TCFC subestimaram o comprimento em 4%.

## INTRODUCTION

Panoramic radiographs are a type of tomography. The structures outside the focal trough are blurred and appear as shadows and artifacts. In order to better maintain the elliptical shape of dental structures within the focal trough, panoramic devices have a center of rotation that changes throughout the scan.^1^ The rotational patterns developed by the manufacturers of these devices vary widely making the resulting images unique to the model. Modifications in arc radius and shape as well as static versus variable centers of rotation have been used to better approximate the shape of the maxillomandibular process in order to maintain patients' dentoalveolar structures within the device's focal trough.^1^ Even with standardized head positions, the great variability in individual's jaw dimensions and shape make achieving optimized panoramic images less predictable and repeatable.

Many reports have noted that panoramic radiographs do not accurately represent tooth positions, thereby requiring the clinician to supplement his findings with a clinical assessment. As reviewed by Van Elslande *et al,* 2 panoramic radiographs are fraught with inconsistent levels of magnification and distortion errors. Some reports^3^ found vertical measurements were ± 10% different from direct measurements of dried skulls, while other groups^4^ found the difference to be as high as 18-21%. Differences in magnification have been found to vary throughout panoramic images. This exacerbated the disparity between devices tested and the majority of manufacturers' documentation which did not accurately correspond to the calculated magnification in various regions of the panoramic images.^2^^,^ 5 While these distortions may be acceptable for ratio calculations, they pose an unacceptable level of unreliability for linear measurements. Turp *et al*'s^6^ analysis of vertical measurements of ramus and condylar heights concurred with Kjellberg's^5^ finding that there was a very low correlation coefficient between the lengths recorded on the panoramic images and direct physical measurements.

Like many clinicians, Kaley *et al* 7 assessed root resorption by comparing pre and post orthodontic treatment panoramic radiographs. The study concluded that a disproportionate number of patients starting with Class III malocclusions and patients with treatment mechanics that positioned maxillary incisor roots in close proximity to the lingual cortical plate had severe root loss. Proclination of incisors to compensate for a Class III malocclusion would have resulted in foreshortening in the panoramic images exaggerating apical resorption. By the same logic, Class II division 1 patients would have underestimated root loss.

Cone-beam computed tomography (CBCT) has offered clinicians a radiographic technique with a high degree of resolution to identify craniofacial landmarks and a spatially accurate means of analyzing them.^8^^,^ 9 ^,^ 10 While CBCT software has the ability to produce panoramic reconstructions, the inherent inaccuracies of conventional image format have prompted only a few studies to compare the accuracy level of these reconstructions not only with conventional images, but also with true anatomy by direct measure. Ludlow *et al* 11 scanned dried skulls with the NewTom 9000 at a resolution of 0.5 mm slice thickness to determine vertical and horizontal length accuracy when reconstructed into panoramic projections. Researchers used metal wires of known length laid along the buccal surface of the ramus and mandibular body as reference knowing that while they likely did not lie in the exact plane of the panoramic reconstruction, as long as they were within 18^o^ of the plane, the foreshortening effect would be less than 5%. Conversely, panoramic reconstruction followed the curvature of the mandible resulting in linear measurements on the image that were overestimated. While operator expertise was considered an important factor in measurement accuracy, the lengths recorded in the 3D volumes by landmark identification in serial axial slices expressed levels of error in the range of 0.19 to 0.37 mm, or 0.6 to 1.7% of the measured lengths. These values were 1.5-2.5 times lower than the panoramic reconstructions of the same volumes. The present study could be considered an extension of Ludlow *et al*'s^11^ project, but instead of using dry skulls, actual patients' data were used. The *in vivo* nature of this study offers orthodontists a clinically realistic result to apply to their diagnosis and treatment planning routines.

The objective of the present study was to determine reliability and accuracy of root length measurements obtained from conventional panoramic radiographs and CBCT panoramic reconstructions, compared to direct root length measurement with digital calipers, considered as the gold standard.

## MATERIAL AND METHODS

The University of Alberta Health Research Ethics Board (Biomedical Panel) approved application #7380 on April 16^th^, 2008. This was a prospective cross-sectional study. Study subjects required maxillary premolar extractions to complete their regular orthodontic treatment goals. The subjects were going to undergo orthodontic treatment. The decision to get a CBCT as well as the need for premolar extractions for the selected cases were generated by the treating orthodontist. Panoramic images needed to be available from previous patient's records. They were not taken in addition to the CBCT imaging. Inclusion criteria for the study required all subjects to have also had conventional panoramic radiograph taken within the previous 24 months. All teeth included in the study were fully erupted maxillary premolars at the time conventional panoramic was taken. All the evaluated premolars appear to have closed apices. CBCT images were taken on the same day the premolars were extracted.

Sample size for the present study was set at 48 teeth. Sample size calculation was performed based on the variability of measurement differences between panoramic images and calipers. Considering the 48 samples as a pilot study, the minimum sample sizes required to identify length differences of 0.5 mm would be 192, and for a 1.0 mm difference, 48. The formula used for this calculation was:



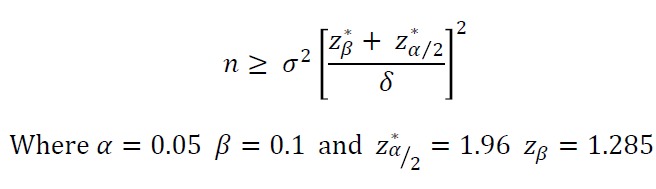



**Figure 1:**
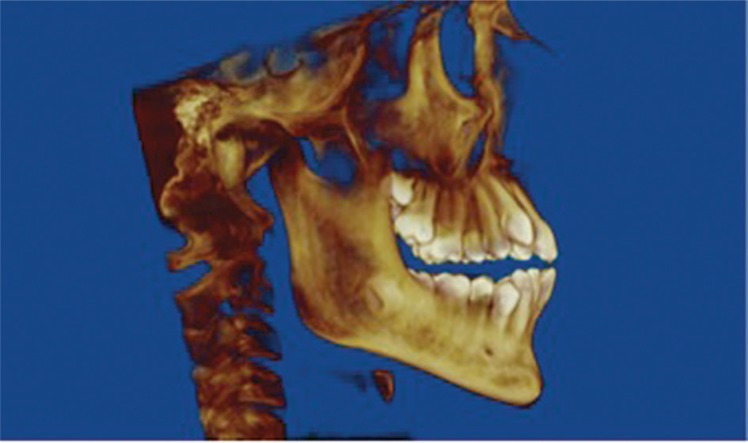
Standardized volume orientation - Sagittal view.

**Figure 2:**
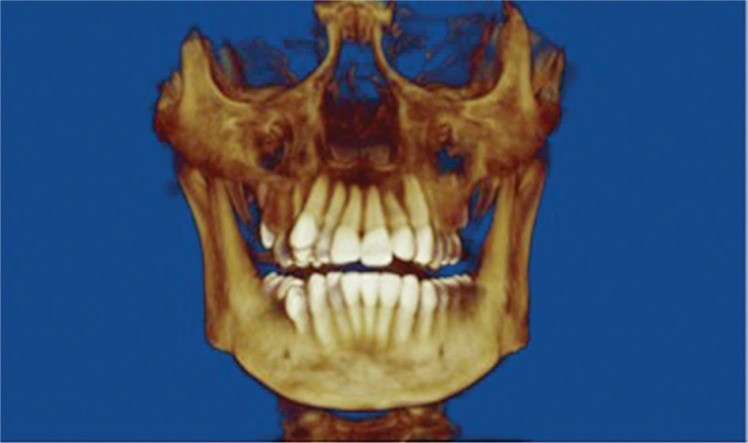
Standardized volume orientation - Frontal View

**Figure 3:**
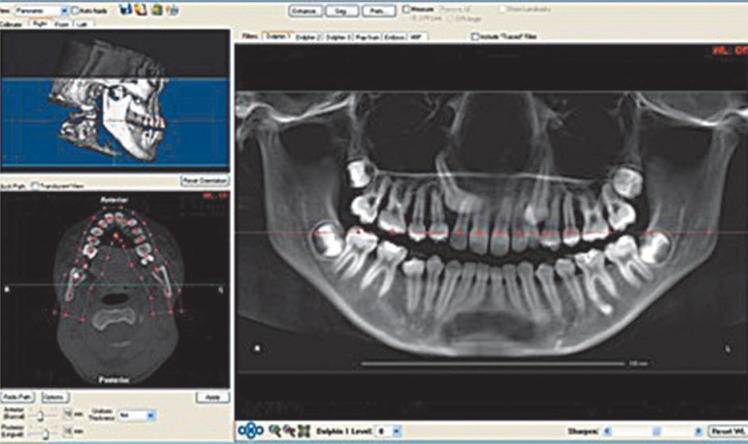
Custom focal trough selection for panoramic reconstruction from CBCT.

**Figure 4:**
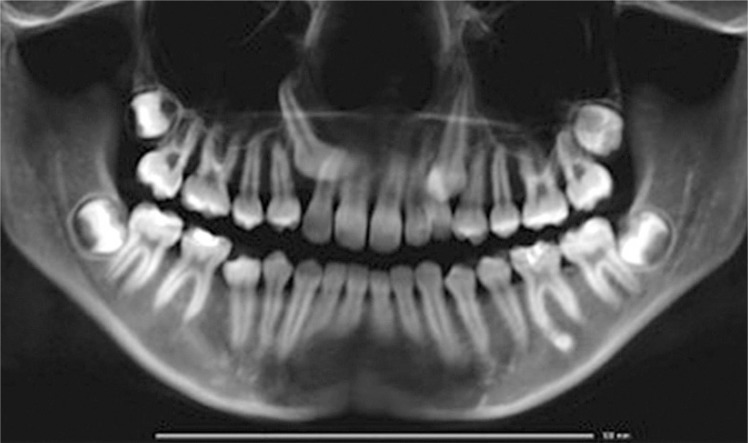
Panoramic reconstruction from CBCT.

CBCT images were taken with the 12-bit i-CAT (Imaging Sciences International, Hatfield, Penn) set to a 40-second scan allowing image reconstruction with a voxel size of 0.25 mm. Standard clinical protocols were used for patients' positioning and a cotton roll between incisor teeth was used to stably hold the occlusion apart to improve cusp tip identification. Images were saved as DICOM files and were reconstructed in Dolphin Imaging 10.5 Premium software (Dolphin Imaging Sciences, Chatsworth, Calif, USA). Head positions in the reconstructed images were standardized anteroposteriorly by Frankfort Horizontal (Fig 1), and sagittally for maximal overlap of bilateral structures in the maxilla, ramus and body of the mandible (Figs 1 and 2) by rotating them spatially. Panoramic images were reconstructed from CBCT volumes by selecting a custom focal trough that passed through the lingual cusps of the maxillary teeth and extended posterior to the condyles. Focal trough width was varied to ensure it encompassed the entire length and height of the maxillary dentition. Axial serial slices were reviewed to ensure the focal trough encompassed all teeth regardless of their angulation and with the center of the custom focal trough bisecting as close to the center of the long axis of the teeth as possible (Figs 3 and 4).

**Figure 5:**
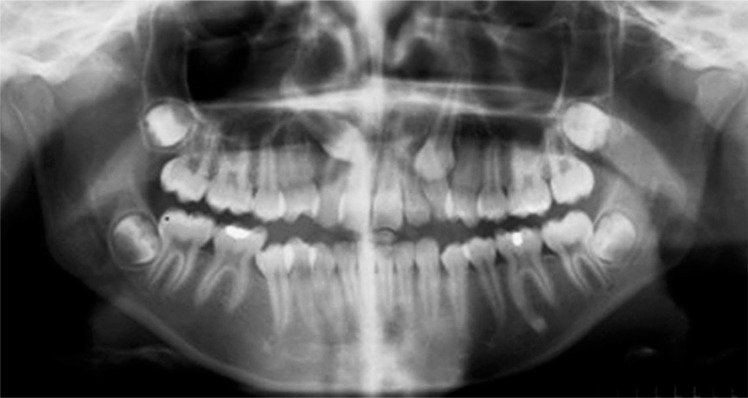
Scanned conventional panoramic radiograph.

Conventional panoramic radiographs were produced with a 17.6 second duration exposure on automatic settings with an Instrumentarium Orthopantomograph OP100 on Fuji Super HRT30 film and Kodak Lanex Regular Intensity screen. The films were developed in a Kodak M35A processor, scanned with an Epson Perfection 700 photo scanner (Epson, Long Beach, Calif) at 300 dpi and 24-bit color, and optimized for contrast and brightness with the Epson scanning software. JPEG images (saved at lowest compression) were imported into Dolphin Imaging for analysis (Fig 5).

Following imaging, one or two maxillary first or second premolar teeth were extracted as per the patient's orthodontic treatment plan and stored in 95% ethanol. The 48 premolars, collected from 26 subjects, were then measured directly with a digital caliper (OrthoPli, Philadelphia, Penn, USA). The minimum caliper reading was 0.013 mm and its measurement accuracy was 0.025 mm as reported by the manufacturer.

The entire tooth length was measured at its longest point from the buccal cusp tip to the root apex. In cases of multiple roots, the buccal root was used unless it had fractured during extraction in which case measurements were made to the intact lingual root apex. These exceptions were noted so the corresponding measurements were made in the panoramic images. Knowledge of dental anatomy was used to assist in the correct identification of buccal cusps and roots in the panoramic images. Consultation with the corresponding extracted teeth was occasionally done to improve the likelihood of correct root selection in situations in which the appropriate root could not be determined due to the tooth's long axis angulation or rotation. Scanned conventional panoramic radiograph measurements were standardized to measurements made on the physical films with the digital caliper. The CBCT panoramic reconstruction measurements were calibrated to the digital ruler produced by the Dolphin Imaging software from the 3D volume. All measurements were recorded to the nearest tenth of a millimeter and done by only one experienced examiner. 

There were no premolars with clinically significant occlusal abrasion. Significant root resorption was not identified in any premolar evaluated as determined in the panoramic image or CBCT generated panoramic image. If any crown abrasion or root resorption happened between images, it was not consider clinically relevant.

### Measurement error

Ten of the 48 samples were randomly selected and measured in triplicate, in random order, with at least one week between each measurement, in order to assess intra-rater reliability. 

### Statistical analysis

Accuracy of tooth length measurements made by the CBCT panoramic reconstructions, conventional panoramic radiographs and the digital caliper (gold standard) were compared to each other by repeated measures one-way ANOVA with Bonferroni correction and by single measures intraclass correlation coefficient using SPSS version 16.0 software (SPSS, Chicago, Ill). Statistical analysis of intra-rater reliability of the triplicate measurements were assessed by single measures intraclass correlation coefficient (ICC) in SPSS.

Statistical analyses for the reliability and accuracy assessments were repeated following the removal of all outlying data points. Since they were determined to have no significant effect on the results, all data points were maintained for the reporting and analyses in this study. Clinically significant changes in root length were considered to be values of 1.0 mm and greater, consistent with those studies by Copeland^12^ and Mohandesan.^13^

## RESULTS

### Reliability

Repeated measures of root length with digital calipers, conventional panoramic radiographs and CBCT panoramic reconstructions had very high reliability with ICC values of 0.999 (95% CI: 0.998, 1.000), 0.997 (95% CI: 0.993, 0.999) and 0.995 (95% CI: 0.995, 0.999) respectively. Landmark identification and thus tooth length measurements were also highly repeatable (intra-observer) in the conventional panoramic images with a single measure ICC of 0.997 (95% CI: 0.993, 0.999) and for the CBCT panoramic reconstructions with a single measure ICC of 0.995 (95% CI: 0.995, 0.999). As another method to verify the degree of reliability, the mean and standard deviation for the differences between the average gold standard tooth length measurements and each corresponding conventional and reconstructed panoramic measurement were also calculated (available upon request).

### Accuracy

Measurements by all three techniques resulted in significantly different tooth lengths (P < 0.001), even when the Bonferroni correction was calculated. The mean tooth length for the conventional panoramic was 6.3 mm (95% C.I.: 5.6 - 7.1 mm) longer than the caliper (gold standard), while the CBCT panoramic mean was 1.6 mm (95% C.I.: 1.1 - 2.0 mm) shorter than the caliper (Table 1).

**Table 1:** Repeated measures ANOVA for measured tooth length with Bonferroni correction.

Orientation (A)	Orientation (B)	Mean difference (A-B)	Significance p	95% CI for difference
Caliper	Conventional Pan	-63	< 0.001	-7.1; -5.6
	CBCT	1.6	< 0.001	1.1; 2.0
Conventional panorex	CBCT Pan	7.9	< 0.001	7.0; 8.8

**Figure 6:**
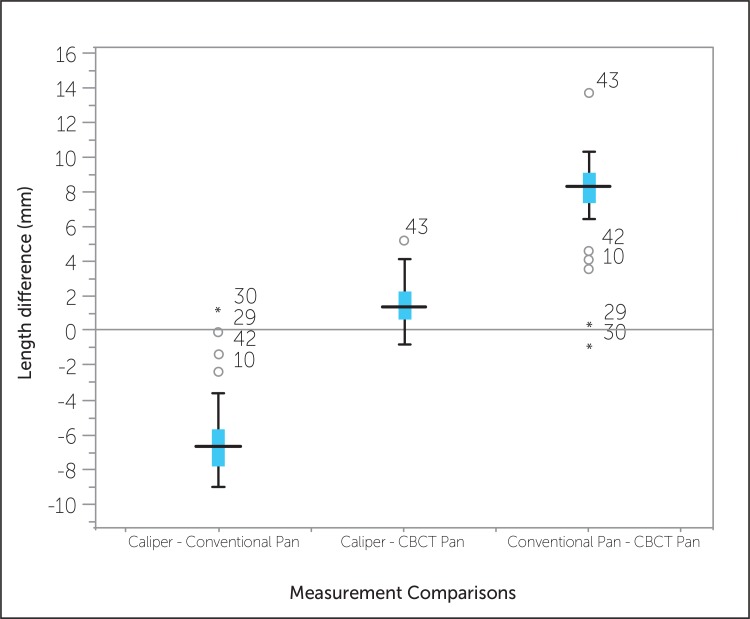
Comparison of tooth length measurements for calipers, conventional panoramic radiographs and CBCT panoramic reconstructions.

Box plots of tooth length differences between the three measurement techniques are depicted in Figure 6. Compared to the caliper (gold standard), the conventional panoramic images resulted in tooth measurements that were generally longer and ranged from 1 mm shorter to 9 mm longer. Tooth lengths in the CBCT reconstructions, on the other hand, were generally shorter than the gold standard, with a smaller measurement discrepancy. These measurements ranged from 1 mm longer to 5 mm shorter than that determined by the calipers. 

**Figure 7:**
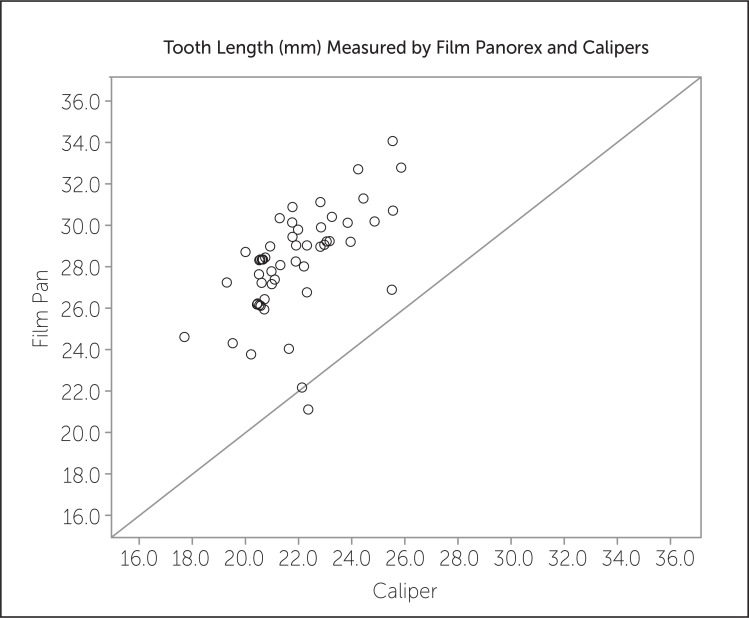
Scatter plot of tooth length - Caliper vs. conventional panoramic

**Figure 8:**
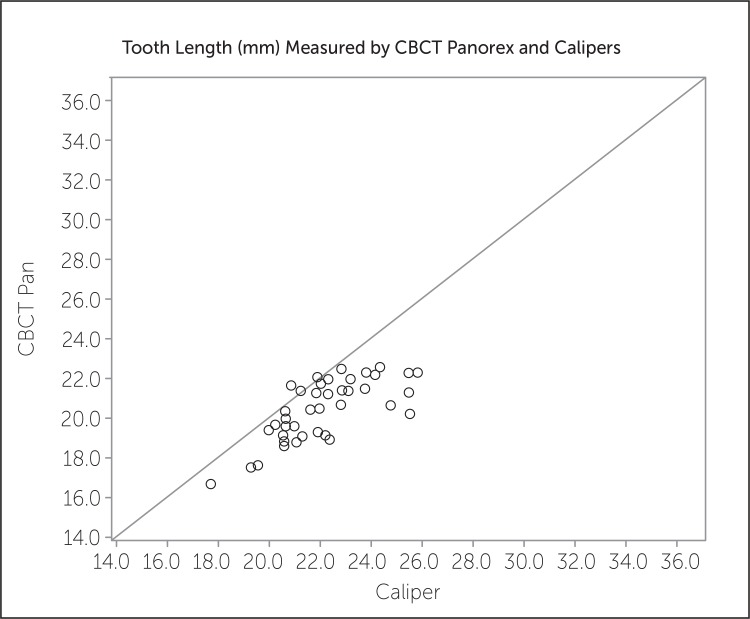
Scatter plot of tooth length - Caliper vs. CBCT reconstructed panoramic.

The 3D CBCT images were standardized for head position sagittally (Fig 1) and coronally (Fig 2) by the by Frankfort Horizontal, inferior orbital rims, condylar heads and inferior border of the mandible prior to panoramic reconstruction. This standardization reduced randomness of elongation and foreshortening distortions compared to the caliper measurements. It did not, however, account for variability in tooth angulation with respect to the standardized neutral head position. Data distribution revealed in the scatter plots (Figs 7 and 8) indicated that tooth lengths showed relatively good measurement reliability across techniques, regardless of actual tooth size, and a measurement bias that resulted in an overestimation of tooth lengths in conventional panoramic images and an underestimation in CBCT panoramic reconstructions. The bias was less distinct for the CBCT reconstructions, as the underestimation appeared to increase for longer teeth (Fig 8).

## DISCUSSION

The average tooth length measured by the conventional panoramic was 6.3 mm (or 29%) longer than the calipers and the range of values was almost twice that of the other measurement techniques. The error in the conventional panoramic measurements in this study is greater than those found by comparison of dry skulls: 10% by Tronje^3^ and 18-21% by Larheim,^4^ but approached the levels of magnification (26%) found by Yitchaky's study.^14^

The average tooth length measured by CBCT panoramic reconstruction was 1.6 mm shorter than the direct measurement by calipers, but the precision of repeated measurements was comparably extremely high for both techniques. This would imply that difference in measured tooth length was not due to misidentification of the landmarks, but to radiographic foreshortening or inadequate resolution of fine root apices compared to the surrounding bone.

A significant limitation of both conventional and CBCT reconstructed panoramic images lies in their inability to account for changes in tooth angulation between serial images when no other assessment means (extra imaging, clinical observation, etc.) are used. During orthodontic treatment, changes in tip and torque introduce elongation and foreshortening errors that cannot be easily accounted for.^15^

It is also possible to mistaken changes in root morphology for resorption as the tooth rotates during treatment and is then projected in only the buccolingual dimension.^7^ An advantage of CBCT reconstructions over conventional panoramic is the ability to more precisely reorient the volumes with the imaging software in order to standardize the image's anatomical planes, thus reducing the error introduced by variable patient position when radiographs are taken by several staff members.^16^ The point of using volumetric imaging should be to actually analyze the information 3D and not to downgrade the image potential during reconstructions. The reason we used reconstructed images was because many clinicians are using them and this study and the discussion should help them better understand the drawbacks.

CBCT images created voxel sizes of 0.25 mm. This translated into a resolution limitation and thus an error of 0.25 mm at each measurement point in the image. Therefore measured tooth lengths from CBCT reconstructions would be expected to achieve accuracy within 0.5 mm of the caliper measurements. 

Although CBCT reconstructions resulted in measurement values that more accurately corresponded to direct caliper measurements compared to those of the conventional panoramic radiographs, it is interesting to note the data patterns that emerged from analysis of the scatter plots (Figs 7 and 8). Conventional panoramic appeared to result in a measurement bias that consistently overestimated tooth lengths regardless of the actual tooth size, whereas CBCT reconstructed images resulted in an underestimation bias that increased for larger tooth sizes. If this bias shows to be consistent, it would allow serial panoramic radiographs to be compared to monitor root changes during orthodontic treatment. Unfortunately, other studies have shown that magnification variability and inherent imaging errors throughout the panoramic images preclude the reliable use of this application.^3^^,^ 4 ^,^ 14

While the tooth length measured from CBCT panoramic reconstructions were statistically and clinically significantly (> 1.0 mm) different from direct caliper measurements, these images provided improved clarity and accuracy compared to the measurements achieved by traditional film panoramic. The underestimation of measurements on CBCT reconstructions compared to direct caliper measurements were consistent with Ludlow's findings^11^ which showed that panoramic reconstructions produced measurement errors of up to 2-4%. The 1.6 mm average decrease in CBCT panoramic tooth length compared to the 22.01 mm caliper mean represented a 7% decrease. With fewer confounding variables compared to conventional techniques, the differences in these measurements were likely due to buccolingual tooth angulation,^15^ and difficulty in landmark identification of cusp and root tips due to tooth rotation, position and anatomy.

### Study limitations

Due to ethical limitations, conventional panoramic radiographs were limited to historical records and, while most were taken within 12 months of the CBCT images and tooth extractions, some records were taken almost 2 years prior to this study. As the patient population was in their early to mid teens, it can be expected that varying amounts of root development would have occurred during the time between conventional and CBCT imaging. While one would expect this to bias the conventional panoramic measurements to be shorter than the caliper and CBCT tooth lengths, the opposite was in fact the case (Fig 7). This indicated that distortion and magnification errors in the conventional images far outweighed any dental growth and apical development.

Several known sources of measurement error in this study were identified. For CBCT panoramic reconstructions, sources such as landmark identification, voxel size resolution limitations, and standardization of the digital and software-generated calipers were addressed and quantified by repeated sample measurements. Others, such as patient positioning variations, focal trough compatibility with patient anatomy, and image artifacts and ghosting were avoided altogether by careful volume positioning and focal trough customization. Sources of error that were not addressed in this study, however, included reliability of standardizing the volume 3D position and focal trough size/shape with the imaging software prior to producing the panoramic reconstructions; and resolution loss accompanying the smoothing, compression and reconstruction algorithms that the Dolphin 3D imaging software used to store and manipulate the large data sets.

An alternative analysis of the data using single measures intra-class correlation coefficient was used to determine the reliability between the three measurement techniques for each tooth individually. The modest ICC value of 0.504 (95% CI: 0.334, 0.660), when calculated with the reliability definition, indicated that the measurement techniques provided only fair agreement in determining if teeth measuring larger by one technique were going to measure larger by the others. Using the absolute agreement definition, however, the very low ICC value of 0.093 (95% CI: -0.016, 0.271) indicated that the magnitude of length differences recorded by one technique did not correspond to equivalent differences in tooth lengths measured by the other techniques. This should be considered when interpreting the present results.

Aspects of the conventional panoramic radiographic technique that were not addressed in this study included variability in patient's head position, imager settings, as well as technician ability and technique, as the images were obtained retrospectively from existing patient records. Ghost images and artifacts from overlapping anatomy are inherent to the conventional imaging process and are not removable to improve dental landmark identification. Additionally, indeterminate levels of magnification and distortion intrinsic in the panoramic images were expected to produce measurement errors not easily accounted for. These may have been exacerbated by focal trough sizes and shapes that did not adequately follow patients' anatomy.

### Clinical implications

Clinicians still must be aware of elongation/foreshortening errors that arise from changes in tip and torque of the teeth of interest when serial panoramic images are compared during treatment. Substantial errors in linear measurement accuracy severely limit conventional panoramic radiography as a tool to identify changes in root length and as such alternative methods should be considered for quantitatively monitoring root resorption. Panoramic reconstructions from CBCT volumes improve measurement accuracy over conventional imaging by reducing several sources of magnification and distortion; however, dental measurements are still significantly different from true anatomical lengths and their use diminishes the accuracy gains achieved by 3D technology. While CBCT panoramic reconstructions provide more reliable representations of changes in tooth length, caution should be exercised when they are used for the diagnosis of early root resorption. 

## CONCLUSIONS

In comparison to actual tooth lengths, conventional panoramic radiographs were relatively inaccurate, overestimating the lengths by 29%, while CBCT panoramic reconstructions underestimated the lengths by 4%.
